# Intragranular strain estimation in far-field scanning X-ray diffraction using a Gaussian process

**DOI:** 10.1107/S1600576721005112

**Published:** 2021-06-14

**Authors:** Axel Henningsson, Johannes Hendriks

**Affiliations:** a Division of Solid Mechanics, Lund University, Lund, Sweden; b School of Engineering, The University of Newcastle, Callaghan, NSW 2308, Australia

**Keywords:** three-dimensional X-ray diffraction (3DXRD), intragranular strain, Gaussian processes, scanning X-ray diffraction

## Abstract

A new method for estimation of intragranular strain fields in polycrystalline materials based on scanning three-dimensional X-ray diffraction (scanning 3DXRD) data is presented and evaluated. Given an *a priori* known anisotropic compliance, the regression method enforces the balance of linear and angular momentum in the linear elastic strain field reconstruction. By using a Gaussian process (GP), the presented method can yield a spatial estimate of the uncertainty of the reconstructed strain field. Furthermore, constraints on spatial smoothness can be optimized with respect to measurements through hyperparameter estimation. These three features address weaknesses discussed for previously existing scanning 3DXRD reconstruction methods and, thus, offer a more robust strain field estimation. The method is twofold validated: firstly by reconstruction from synthetic diffraction data, and secondly by reconstruction of a previously studied tin (Sn) grain embedded in a polycrystalline specimen. Comparison against reconstructions achieved by a recently proposed algebraic inversion technique is also presented. It is found that the GP regression consistently produces reconstructions with lower root-mean-square errors, mean absolute errors and maximum absolute errors across all six components of strain.

## Abstract 2

A novel regression method for estimating intragranular strain in polycrystalline materials from three-dimensional X-ray diffraction data is presented and evaluated. The method incorporates an equilibrium constraint to the reconstructed strain field by using a Gaussian process.

## 1. Introduction   

Three-dimensional X-ray diffraction (3DXRD), as pioneered by Poulsen (2004 ▸) and co-workers, is a nondestructive materials probe for the study of bulk polycrystalline materials. The experimental technique is typically implemented at synchrotron facilities where access to hard X-rays (≥10 keV) facilitates the study of dense materials with sample dimensions in the millimetre range. In contrast to powder diffraction, 3DXRD enables studies on a per-grain basis, which requires that the Debye–Scherrer rings consist of a set of well defined, separable single-crystal peaks. To achieve this, the beam and sample dimensions must be selected accordingly, limiting the number of grains illuminated per detector readout. By various computer-aided algorithms (*cf.* Lauridsen *et al.*, 2001 ▸), the single-crystal diffraction peaks can be segmented and categorized on a per-grain basis, enabling the study of individual crystals within a sample. Typical quantities retrieved from such analyses are the grain average strain and average orientation (Poulsen *et al.*, 2001 ▸; Oddershede *et al.*, 2010 ▸). From further analysis it also possible to retrieve an approximate grain topology map (Poulsen & Schmidt, 2003 ▸; Poulsen & Fu, 2003 ▸; Markussen *et al.*, 2004 ▸; Alpers *et al.*, 2006 ▸).

Reducing the X-ray beam cross section to sub-grain dimensions not only allows for the study of samples with large numbers of grains but also enables the investigation of intragranular variations. This special case of 3DXRD is commonly referred to as scanning 3DXRD since, to acquire a full data set, the narrow beam must be scanned across the sample. In this setting, it is possible to measure the diffraction signal from approximate line segments across the grains, collecting information on the intragranular structure. Any inversion procedure, in pursuit of such intragranular quantities, then poses a rich tomography problem where the ray transform typically involves higher-order tensorial fields.

Recent advances in diffraction contrast tomography (Reischig & Ludwig, 2020 ▸) show promising results for inversion for both orientation and strain fields in three dimensions with intragranular resolution. In scanning 3DXRD where higher angular resolution on scattering vectors is achieved at the cost of diffraction peak resolution (Nervo *et al.*, 2014 ▸), multiple proposals for inversion operating solely from scattering vectors have been made. Initially, Hayashi *et al.* (2015 ▸, 2017 ▸) proposed a method for a per-voxel strain refinement to approximate intragranular strains using scanning 3DXRD data. Unfortunately, this procedure was shown to introduce bias in the reconstruction related to strain state (Hayashi *et al.*, 2019 ▸; Hektor *et al.*, 2019 ▸). These obstacles were later overcome by Henningsson *et al.* (2020 ▸), who proposed an inversion method that takes the tomographic nature of the problem into account. As has been pointed out by several other authors (*cf.* Margulies *et al.*, 2002 ▸; Lionheart & Withers, 2015 ▸), the sampling of strain is not uniform in 3DXRD and, as a result, some additional constraints on the reconstructed field are often desirable. Henningsson *et al.* (2020 ▸) proposed a simple smoothing constraint to each of the strain components with success. However, the parameter selection and the physical interpretation of these constraints are not well defined.

For powder-diffraction-type data, excellent progress to overcome the weaknesses highlighted above has been made using a Gaussian process (Hendriks *et al.*, 2020 ▸). In this current work, we adapt the Gaussian process (GP) framework to scanning 3DXRD and extend it to a wider class of anisotropic materials. This framework allows for the introduction of a static equilibrium constraint, which ensures that the retrieved strain reconstruction will satisfy the balance of both angular and linear momentum. The GP naturally incorporates spatial correlation in the predicted fields via a covariance function, which, together with the equilibrium prior, replaces the need for previously used smoothing constraints. Moreover, the GP produces an estimate of the uncertainty in the reconstructed strain field, as a by-product of regression. Overall, the presented regression procedure addresses several weaknesses of previous work and provides a tool for uncertainty estimation in the reconstructed strain fields.

## 2. Diffraction measurements   

### 2.1. Experimental acquisition   

**Figure 1:**
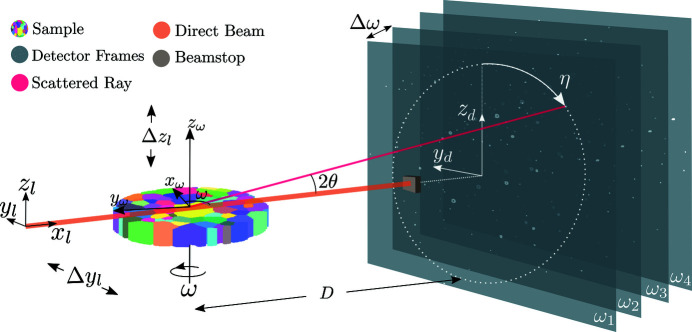
Scanning 3DXRD experimental setup. The sample coordinate system (subscript ω) is attached to the sample turntable while the laboratory (subscript l) coordinate system is fixed in relation to the sample. The sample is rotated and translated in the *y*_l_ *z*_l_ plane across the beam to record diffraction from the full volume [modified from Henningsson *et al.* (2020 ▸)].

In scanning 3DXRD, a polycrystalline specimen is placed on a sample stage associated with an attached coordinate system (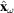, 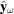, 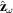). The sample stage commonly has several degrees of freedom, some of which are used for initial alignment and calibration and others for data collection. Since the calibration procedure is the same for all 3DXRD-type experiments, here we only describe the degrees of freedom related to data acquisition; for details on calibration see Oddershede *et al.* (2010 ▸), Edmiston *et al.* (2011 ▸), Borbely *et al.* (2014 ▸) and Sharma *et al.* (2012 ▸). A fixed laboratory coordinate system (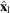, 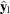, 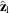) is introduced, which is related to the sample coordinate system through a positive rotation about 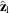 and a translation in the 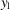 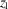 plane (Fig. 1 ▸). For a given sample position (*y*_l_, *z*_l_), rotation in ω is performed in discrete steps of Δω. The scattered intensity in each Δω rotation interval is generally integrated during the acquisition, resulting in a series of frames for each (*y*_l_, *z*_l_) position. After any necessary spatial distortion corrections have been made, the raw pixelated image stacks (*y*_d_, *z*_d_, ω) can be segmented into separate connected regions of diffracted intensity for which centroids and average intensities can be calculated. The resulting data set is six dimensional, with each diffraction peak average intensity and detector centroid (θ, η) mapping to a sample stage setting (*y*_l_, *z*_l_, ω).

### 2.2. Laue equations and scattering notation   

From the diffraction peak centroids (θ, η) it is possible to compute scattering vectors, **G**, defined in the laboratory frame as 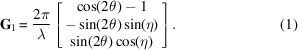
Using the notation of Poulsen (2004 ▸) and considering that the Laue equations are fulfilled during diffraction, we can also express the scattering vectors as 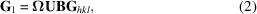
where **Ω** and **U** are unitary square 3 × 3 rotation matrices describing, respectively, the turntable rotation around 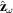 and the crystal unit-cell orientation with respect to the ω-coordinate system. The matrices **U** and **B** can now be uniquely defined as the polar decomposition of their inverse product, (**U** **B**)^−1^, in which the rows contain the real-space unit-cell lattice vectors **a**, **b** and **c** described in the sample ω-coordinate system:
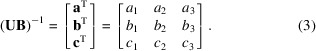
The integer vector **G**_*hkl*_ = [*h* *k* *l*]^T^ holds the Miller indices.

### 2.3. Grain mapping   

**Figure 2:**
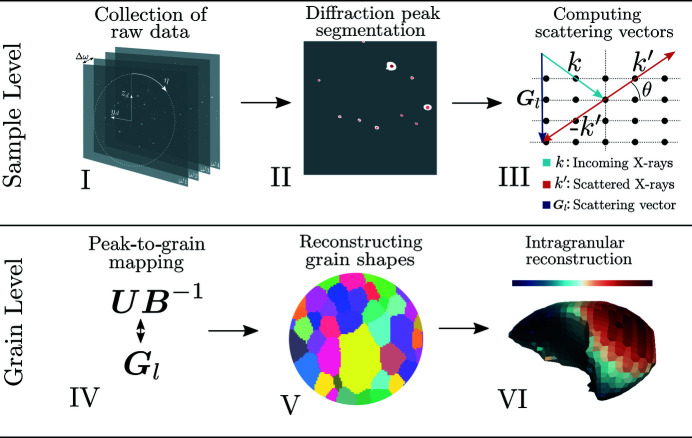
Simplified schematic of analysis steps commonly performed on scanning 3DXRD data. From raw detector data (I), the per-peak centroids (η, θ) and average intensities are retrieved (II). The scattering vectors can then be computed (III) and input into a peak-grain mapping algorithm (IV). From the produced maps, per-grain shape reconstruction can take place (V). Finally, intragranular quantities can be sought (VI).

Given a measured set of scattering vectors, the procedure known as grain mapping is concerned with finding a set of uniform crystals that explain the data. In this setting, grains are represented by their average (**U** **B**)^−1^ matrices together with their real-space centroid coordinates. To contextualize the grain-mapping procedure, a simplified schematic of the scanning 3DXRD analysis steps is presented in Fig. 2 ▸.

In essence, the grain-mapping procedure results in a map between diffraction peaks and individual average grain (**U** **B**)^−1^ matrices and centroids. The diffraction peaks associated with a single grain can be extracted from such peak–grain maps and grain shape reconstruction can be performed by tomographic methods (*cf.* Poulsen & Schmidt, 2003 ▸; Alpers *et al.*, 2006 ▸), utilizing the scattered intensity associated with each diffraction peak. The peak–grain maps also enable studies on a per-grain basis, something which simplifies analysis both conceptually and computationally. Software for performing grain mapping is freely available in the *ImageD11* package (Wright, 2005 ▸), and further details on various algorithm options can be found in the literature (Moscicki *et al.*, 2009 ▸; Oddershede *et al.*, 2010 ▸; Edmiston *et al.*, 2011 ▸; Sharma *et al.*, 2012 ▸; Schmidt, 2014 ▸). In this paper we are concerned with reconstruction of intragranular strain, and thus we focus on the final step of analysis and proceed with the assumption that all preceding quantities have been computed.

## 3. Measurement model   

### 3.1. Strain revealing transformations   

Henningsson *et al.* (2020 ▸) described the procedure to calculate strains in individual lattice planes from scanning 3DXRD measurements via the Bragg equations as first laid out by Poulsen *et al.* (2001 ▸) and Margulies *et al.* (2002 ▸). To enrich the framework, allow for consistent use of the Laue equations and clarify how the integration of strain can take place, here we adopt a different route, rewriting the Laue equations and performing a first-order Taylor series expansion. We start by recollecting that the 3 × 3 continuum deformation gradient tensor, **F**, should have the property that 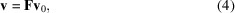
where **v**_0_ is a vector in the reference configuration and **v** is the corresponding deformed vector. Applying this to a crystal reference unit cell (**a**_0_, **b**_0_, **c**_0_) given in the sample ω-coordinate system and collecting the equation in matrix format, we find that 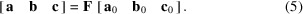
With (3) this allows us to identify that 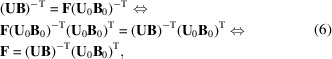
where **U**_0_ and **B**_0_ define an undeformed crystal lattice. We can now relate the quantities involved in the Laue equations (1) to the strain tensor by considering that the infinitesimal strain tensor in the sample ω-coordinate system is defined as 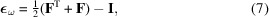
where **I** is the identity tensor. An introduction to elasticity theory is provided by Ottosen & Ristinmaa (2005 ▸). Insertion of (6) into (7) gives 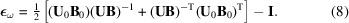
The observable quantity in 3DXRD is the scattering vectors and a useful formulation must therefore relate **ε**_ω_ to **G**_ω_, together with the known quantities **U**_0_ and **B**_0_. To achieve this we consider the strain in a single direction, introducing the unit vector 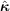 into (8) as 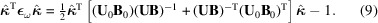
The problem is now to select 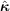 such that the right-hand side reduces to an observable quantity. From (2) we may define 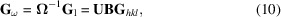
and sample the strain parallel to this scattering vector as 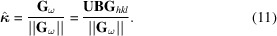
Insertion into (9) now reduces (9) to 
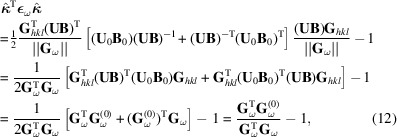
where 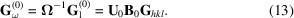
This selection of unit vector 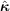 not only guarantees that **ε**_ω_ is the only unknown in (12) but further spreads the sampling of strain to all directions defined by the measured set of scattering vectors **G**_ω_. For high X-ray energies, although not uniform, this spread is typically good (Lauridsen *et al.*, 2001 ▸), explaining why, in general, strain reconstruction is possible in 3DXRD.

### 3.2. Tensorial ray transform   

So far we have worked with equations (2)–(12) as if scattering occurred from a single point. This is typically the approximation made in 3DXRD when only grain average properties are required. For scanning 3DXRD, when pursuing intragranular quantities, we must consider that scattering takes place from grain sub-regions, illuminated by the narrow X-ray beam. In fact, if the scattered intensity is the same from all points within the grain, the scattering vectors known to us from the experiment are average quantities over regions, 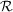, within the grain such that 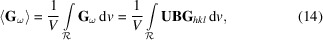
where *V* is the total volume of 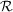, d*v* is the differential on 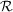 and 〈·〉 indicates volume average. We run now the risk of invalidating our previous result (12) since the local scattering vectors **G**_ω_ = **G**_ω_(*x*_ω_, *y*_ω_, *z*_ω_) are unknown in scanning 3DXRD. To maintain a useful expression we must further transform (12) into an equation in 〈**G**_ω_〉 rather than **G**_ω_. However, since the strain is nonlinear in **G**_ω_, direct volume integration of (12) is not possible. Fortunately though, we may obtain an approximation by Taylor expansion of (12) at 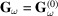 to first order: 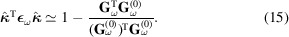
By selecting a uniform reference configuration in space, integration of (15) now gives, with (14), that 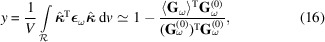
where we introduce the scalar average strain measure 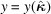.

Finally, in any inversion scheme where **ε**_ω_ constitute the free variables, we must be able to execute the forward model that is the integral of (16). For this purpose the direction of strain, 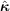, must be approximated. Using the already introduced assumption that **G**_ω_ varies weakly over 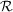 we can write 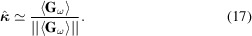
We note that, equally, the approximation 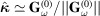 could have been made.

In conclusion, (16) and (17) relate the measured average scattering vectors, 〈**G**_ω_〉, to the underlying strain field, **ε**_ω_(*x*_ω_, *y*_ω_, *z*_ω_), with the strain tensor being the only involved unknown quantity.

The approximations made in (16) and (17) will give rise to an error in the integrated strain value *y*. The magnitude of this error will strongly depend on the spatial distribution of intragranular strain and orientation. To demonstrate that the approximations made in (16) and (17) are accurate for small strains and moderate mosaic spreads, we provide an extended analysis of this error in Appendix *A* [App appa]. This discussion also highlights why, and when, it is possible to reconstruct intragranular strain independently of intragranular orientation in scanning 3DXRD.

### 3.3. Estimated uncertainty   

To finalize the measurement model we introduce an additive Gaussian error *e* into (16), representing measurement uncertainty. Furthermore, to simplify both computation and further analytical derivations we approximate the volume integral over 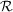 by a corresponding line integral going through the geometrical centre of this region. Finally, we have the measurement model 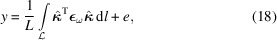
where *L* is the length of the line segment 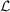, d*l* is the differential on 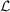 and *e* is the additive normally distributed noise: 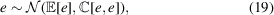
with expectation value 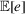 and covariance 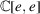.

The measurement noise is assumed to be zero mean (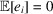) and independent (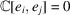) with the variance selected in accordance with previous work (Borbely *et al.*, 2014 ▸; Henningsson *et al.*, 2020 ▸), 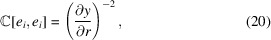
where *r* = *r*(θ) is the radial detector coordinate and the indices *i* and *j* indicate unique measurements. Other estimations of 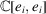 are possible. Importantly, though, the variance should depend on the scattering angle 2θ, as, for a 2D detector with uniform pixel size, the measurement uncertainty increases with decreasing scattering angle.

## 4. Regression procedure   

Equation (18) is a ray transform that contains information on the average directional strain for a region within the grain. The problem to reconstruct the full strain tensor field from a series of such measurements is therefore tomographic in nature, and the measurements *y* are highly spatially entangled as the regions 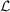 will intersect in general. A collection of *N* measurements, 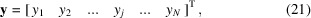
could represent the second member of a linear equation system where (18) is used to form a system matrix and a vector of unknown strains defined on some finite basis. This has been described by Henningsson *et al.* (2020 ▸) for a voxel basis, using a weighted least-squares (WLSQ) approach to retrieve the strain field. As we will discuss in Section 4.1, in this work we adapt these ideas to a Gaussian process framework, not solving for a deterministic strain field but instead calculating the probability distribution of strain at each spatial coordinate, revealing a distribution over strain tensor functions.

**Figure 3:**
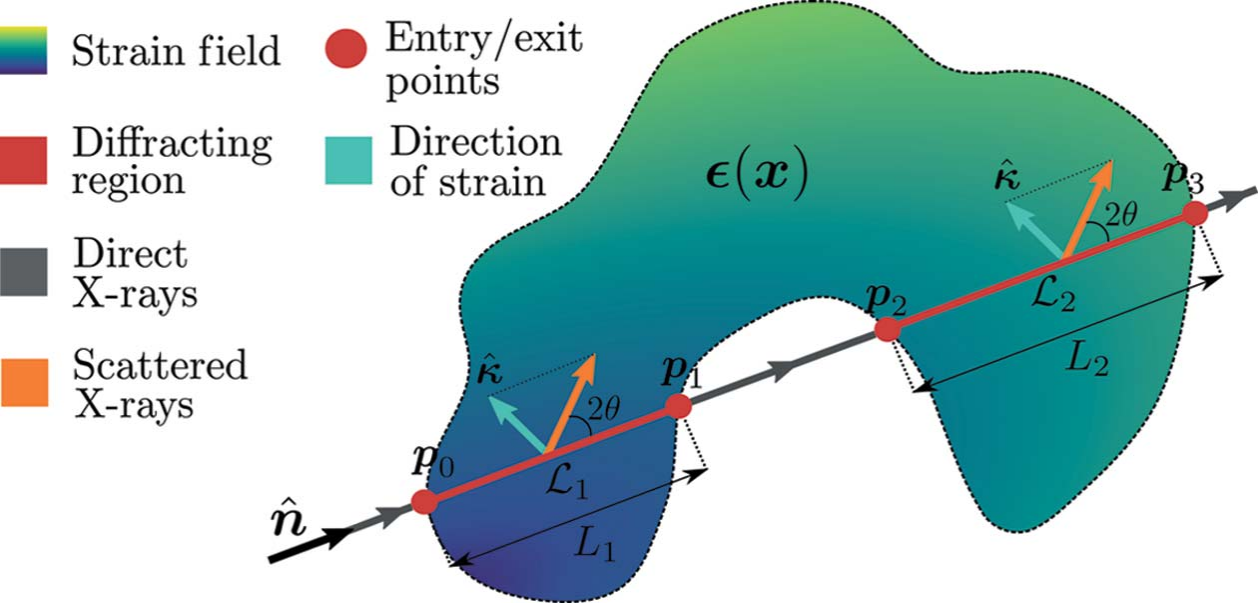
A single crystal under elastic deformation illuminated by an X-ray beam. Scattering takes place along the illuminated region 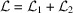.

Before proceeding any further, it is useful to introduce a vector notation along with some geometrical quantities related to the integration path 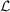 (Fig. 3 ▸).

Since **ε** ^T^ = **ε** we can uniquely represent the strain tensor field in sparser format by introducing the column vector 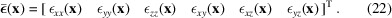
To represent the tensor product 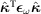 involved in (18) using 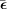 we seek the corresponding vector 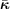 such that the equality 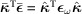 holds true. We find by expansion that 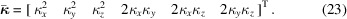
Next, denoting the intersection points between the X-ray beam and the grain boundary by **p**_0_, **p**_1_,…, **p**_*M*_ and letting the Euclidean length of these illuminated regions be labelled *L*_*i*_ = ||**p**_*i*_ − **p**_*i*+1_||_2_, we find, for measurement number *j*, that 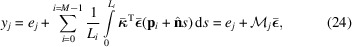
where the symbol 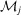 is shorthand for the integral operator corresponding to measurement number *j*, *s* is a scalar, 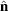 is a unit vector along the X-ray beam and 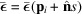 is a function over a compact support in the grain volume. Considering the full measurement set **y** defined in (21), we introduce a compact notation, 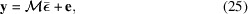
where 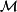 and **e** are column vectors formed in analogy with (21).

### 4.1. Gaussian process regression   

A Gaussian process is any stochastic process in which all subsets of a generated stochastic sequence of measurements form multivariate normal distributions (Rasmussen, 2003 ▸). The regression procedure associated with a Gaussian process, known as Gaussian process regression, can be described in terms of basic statistical theorems and quantities. The central idea is to exploit the fact that linear operators acting on normally distributed variables form again normal distributions. The goal is to arrive at the distribution of the Gaussian process that, for some spatial function *f*(**x**), describes the probability of finding a value *f* at coordinate **x** together with the covariance of *f*(**x**) with other spatial locations *f*(**x**′).

In the scanning 3DXRD case, we consider the measurement series, **y**, generated by some underlying strain tensor field, 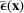, and seek to calculate at each spatial coordinate, **x**, the probability distribution 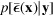, *i.e.* the probability of finding a specified strain tensor 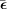 at **x** given the measurements **y**. As we will show, if we assume a Gaussian process prior and Gaussian noise, this probability distribution is multivariate normal, and the covariance of strain at any two points, 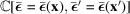, together with the strain expectation value, 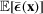, will be revealed by the regression.

If it is assumed that 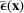 is normally distributed, 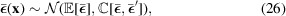
it follows directly that **y** is multivariate normal, 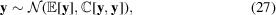
since it is a linear combination of the independent normal distributions 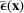 and **e**. Considering then the joint distribution of 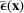 and **y** we can calculate 
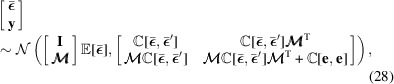
where **I** is an identity operator and we use the fact that **y** is a linear transformation of two normally distributed variables 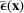 and **e**. The joint probability of (28) now gives us the sought distribution, 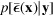, which is again normal. Its variance and expectation value can be found by writing out (28) in analytical exponent form, with fixed **y**, and completing the exponent square. The closed-form solution can be obtained as
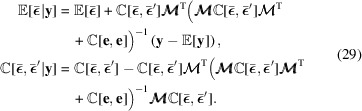
Before any approximate or analytical solutions to the involved transformations of 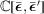 by 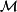 can be given, it remains first to specify the prior distribution of 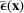.

### 4.2. Equilibrium prior   

Since the closed-form solution of (29) requires only that 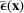 is normal, we are free to incorporate prior knowledge on 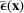 by making a parametrization of 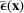 as linear transformations of some other underlying normal distributions. Since 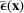 represents a linear elastic strain field and the scanning 3DXRD experiment is assumed to take place on a sample at rest, we expect that the accompanying stress field 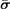 will be in static equilibrium. This can be expressed as a linear map 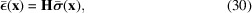
where **H** is an anisotropic compliance matrix that is orientation dependent, 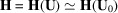. The set of analytical functions 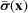 that satisfy balance of angular and linear momentum are known as the Beltrami stress functions. These may be described as a linear map 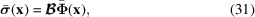
where 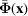 is a column vector holding six Beltrami stress functions, which are required to be twice differentiable, and 
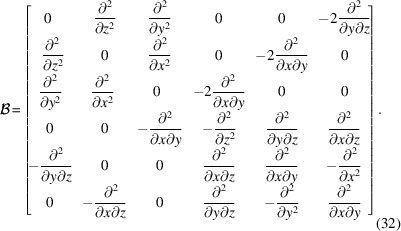
We have then
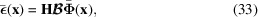
and must now make an assumption on the distribution of 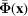. Without any further prior knowledge we select a zero-mean normal distribution with 
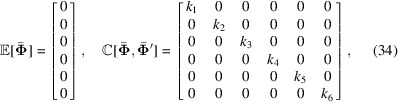
where the covariance functions 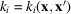 describe the spatial correlation of the field. In this work, we have used the stationary squared-exponential covariance function, 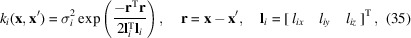
introducing a smoothness assumption into the strain field reconstruction. The unknown hyperparameters defined by 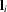 and σ_*i*_ are thus in total 6 × 4 = 24 in our case. These variables will be estimated through an initial optimization process known as hyperparameter optimization; we will return to how this is done later. First we highlight that the zero-mean prior assumption on the Beltrami stress functions, 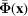, does not imply that the posterior distribution of strain, 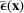, will be zero mean. This is realized upon examination of equation (29), which shows that a prior mean of 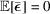 does not imply that the conditional posterior 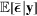 will be zero. Other selections for the prior mean are possible; however, when such additional prior information is unknown, a zero-mean selection is preferable for simplicity.

In total, these selections impose that (i) the strain field is in a point-wise static equilibrium and (ii) the strain field has a local spatial correlation to neighbouring points. The resulting prior distribution of strain is 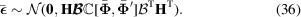



### 4.3. Equilibrium posterior distribution   

With the prior information of equilibrium and spatial correlation now encoded into the strain field we can insert 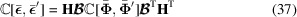
into equation (29) to arrive at a final expression in which only the hyperparameters remain to be estimated. The covariance between measurements takes on the form 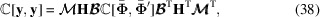
which involves, through the mappings 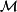, a double integral over the two times partially differentiated squared exponential in (35). The solution to this double line integral is intractable, although some work has been done to show that for *l*_*x*_ = *l*_*y*_ = *l*_*z*_ it can be analytically reduced to a single integral (Hendriks, Gregg *et al.*, 2019 ▸). However, the numerical integration remains too computationally costly for practical use. This motivates the use of an approximation scheme on a reduced basis for which closed-form solutions to all involved quantities of (29) are again recovered (Jidling *et al.*, 2018 ▸).

### 4.4. Finite basis approximations   

Decomposing (35) onto a Fourier basis, 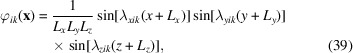
where the scalars λ and *L* are the frequencies and phases, respectively, we find that 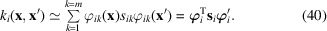

**s**_*i*_ is a diagonal matrix of basis coefficients, *s*_*ik*_, which are the spectral densities of (35). Specifically it is possible to show (Solin & Särkkä, 2020 ▸) that the *k*th spectral density is 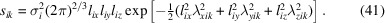
With the vector notation 
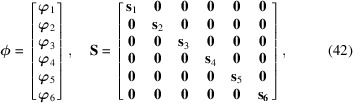
where **0** is a matrix of zeros, we find the approximate covariance
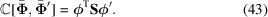
Insertion of (43) into (37) now yields 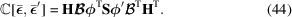
Introducing the quantities 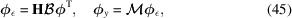
we finally arrive at the approximate posterior mean and covariance of strain using (29):
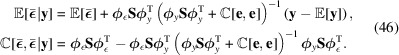
The computational complexity can be further reduced by algebraically rearranging this equation to avoid forming the covariance matrices (Rasmussen, 2003 ▸), resulting in 
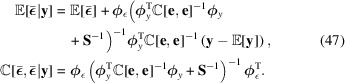
Here, the inverses **S** ^−1^ and 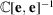 can be trivially computed, as the matrices are diagonal. For *m* < *N*, this reduces the computational complexity to 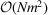 from 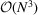 required for the inverse in (29) and (46). A numerically stable and efficient algorithm for solving these equations using the QR decomposition is given by Hendriks, Wensrich *et al.* (2019 ▸), together with analytical expressions for the various integral mappings 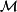. We note here that, although the introduced Fourier basis in (39) is defined over all space, the support of the reconstructed field in (47) is for all practical purposes that of the grain volume. This follows from the fact that the mappings executed through 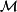 are only performed over the grain, as indicated in (24), and requires that the period of the lowest frequency basis included is larger than the grain volume.

As *m* → ∞ the approximate solution (47) approaches the exact solution (29) (Solin & Särkkä, 2020 ▸). In practice, however, we must select a finite *m*, leading to (35) being used in approximate form. To direct the selection of frequencies λ_*xik*_, λ_*yik*_ and λ_*zik*_ in (40) use can be made of (41). In this work, we have selected the basis frequencies on an equidistant grid in (λ_*xik*_, λ_*yik*_, λ_*zik*_) space such that the spectral densities were above a minimum threshold, *i.e.* we aim to achieve a desired coverage of the spectral density function. Specifically, we select 
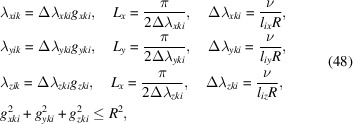
where (*g*_*xki*_, *g*_*yki*_, *g*_*zki*_) are positive integers such that (Δλ_*xki*_ *g*_*xki*_, Δλ_*yki*_ *g*_*yki*_, Δλ_*zki*_ *g*_*zki*_) defines equidistant grid points excluding the origin, and ν controls the desired coverage of the spectral density.

To see how ν controls this coverage, we use equation (48) to write the spectral density in (41) as a function of ν, giving 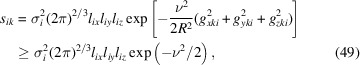
where the inequality holds because the maximum value of (*g*_*xki*_, *g*_*yki*_, *g*_*zki*_) is *R* ^2^. Hence, we can see that ν controls the minimum spectral density, or alternatively we could view it as controlling the proportion of the volume under the spectral density function we wish the basis functions to cover. Taking this view, ν = 1 gives ∼68%, ν = 2 gives ∼95% and ν = 3 gives ∼99.7% volume coverage. In this work, we use ν = 3.5, corresponding to approximately 0.9996% coverage of the volume under the spectral density function.

Continuing with this reasoning, we can view *R* as governing the resolution with which the basis functions cover the spectral density. Whilst larger *R* will result in a better approximation to the covariance function it also increases the computational cost and, in general, will have diminishing returns in terms of error reduction. A suggestion is to increase *R*, subject to computational limits, whilst observing a substantial reduction in residuals or improvement in the out-of-sample log likelihood – described in detail in the next section. For both the simulation and real data experiments in this work we have used *R* = 5, which results in a total of *m* = 38 used basis functions for each of the six covariance functions, *k*_*i*_(**x**, **x**′), *i* = 1, 2,…, 6. Increasing *R* beyond this was found to give minimal improvement.

To complete the regression scheme, we now discuss the selection of the hyperparameters *l*_*ix*_, *l*_*iy*_, *l*_*iz*_ and σ_*i*_, which at this stage are the only unknowns in the formulation.

### 4.5. Hyperparameter selection   

The hyperparameters, *l*_*ix*_, *l*_*iy*_, *l*_*iz*_ and σ_*i*_, for the posterior conditional distribution can be determined through optimization (Rasmussen, 2003 ▸). Typically, this is done by either maximizing the log marginal likelihood or using a cross-validation approach and maximizing the ‘out-of-sample’ log likelihood, *i.e.* the likelihood of observing a set of measurements not used in the regression, 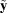. Following the work by Gregg *et al.* (2020 ▸), which demonstrates that maximizing the out-of-sample log likelihood yields better results for line integral measurements, we determine the hyperparameters by solving 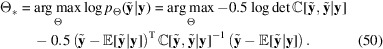
where Θ is a vector holding the hyperparameters introduced in (35) and 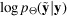 is the out-of-sample log likelihood. By extension of (47), we have that 
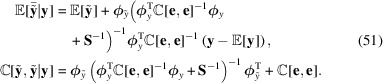



Note that it is not essential that a global optimum is found in this procedure; in fact, in many cases, setting the hyperparameters to some reasonable fixed values will produce excellent reconstructions. In the case of scanning 3DXRD we have found that setting the hyperparameters uniformly to the grain diameter gives reasonable results and can serve as a good initial guess for optimization.

## 5. Validation   

To validate the presented regression method we have generated simulated scanning 3DXRD data using a previously developed algorithm (Henningsson, 2019 ▸). This tool has been used with success in the past (*cf.* Hektor *et al.*, 2019 ▸; Henningsson *et al.*, 2020 ▸) and can provide an understanding of the limitations and benefits of scanning 3DXRD reconstruction methods. Briefly, the simulation input is specified as a set of cubic single-crystal voxels featuring individual strains and orientations together with an experimental setup. We refer the reader to Henningsson (2019 ▸) for additional details on the simulation algorithm, with an undocumented implementation available via https://github.com/FABLE-3DXRD/S3DXRD/. Strain reconstructions from generated diffraction data were compared with ground-truth input strain as well as an additional reconstruction method described by Henningsson *et al.* (2020 ▸). This reconstruction method, previously referred to as ‘algebraic strain refinement’ (ASR), uses a voxel basis for strain reconstruction and can, in short, be described as solving a global WLSQ problem. This least-squares approach operates from the same average directional strain data as the presented GP method.

### 5.1. Single-crystal simulation test case   

**Figure 4:**
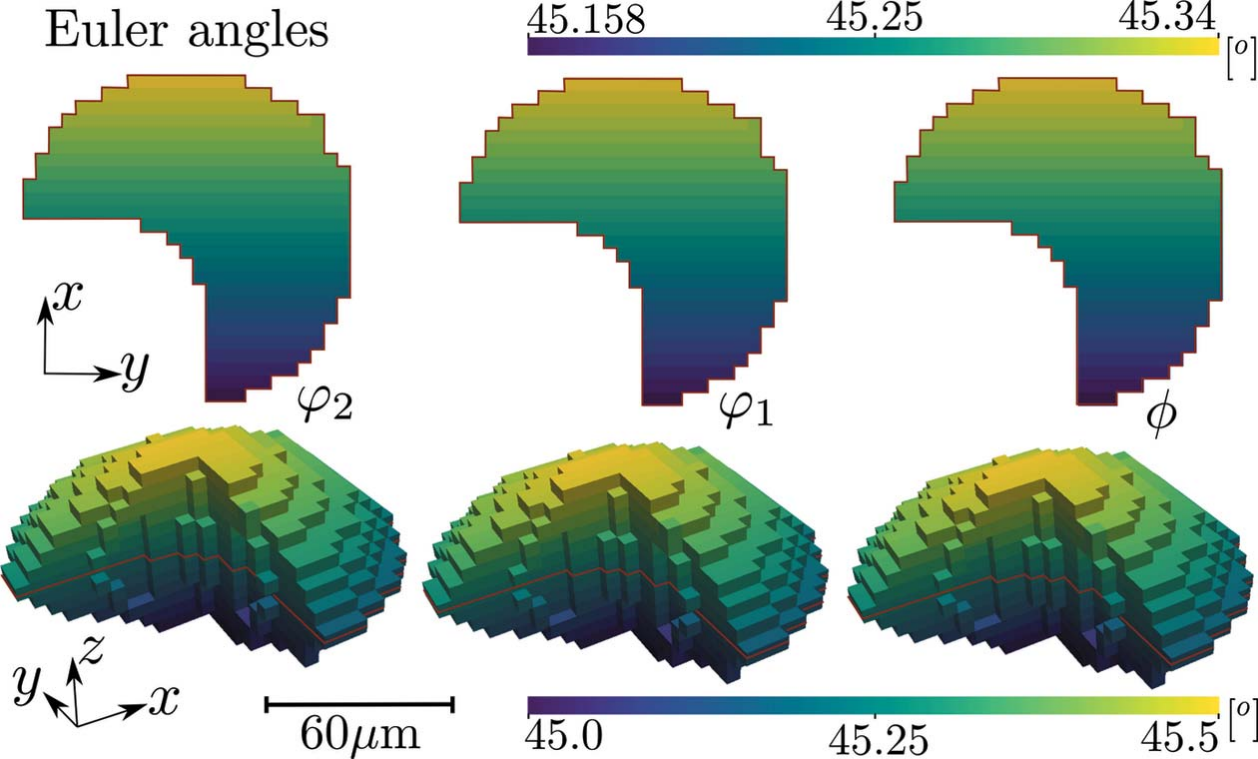
Grain topology input for diffraction simulation coloured by corresponding input Euler angle field in units of degrees. The top row represents central cuts through the 3D renderings below, as indicated by the red lines.

Diffraction from a tin (Sn) grain subject to a nonuniform strain tensor field has been simulated for the nonconvex grain topology depicted in Fig. 4 ▸.

The grain was assigned an orientation field by introducing linear gradients in the three Euler (Bunge notation) angles, φ_1_, Φ, φ_2_, as 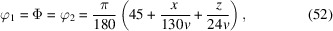
where *v* = 5 µm was the used voxel size and the grain origin was set at the grain centroid in the *xy* plane and at the bottom edge of the grain in *z* (Fig. 4 ▸). The maximum grain size in each dimension *x*, *y* and *z* was 26, 26 and 13 voxels, respectively.

The strain field was defined by a set of Maxwell stress functions, which are a subset of the more general class of Beltrami stress functions, 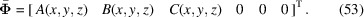
To achieve a relatively simple, but not trivial, strain field the functions *A*, *B* and *C* were selected as a cubic polynomial:
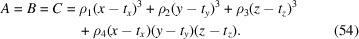
The stress was converted to strain by the elastic compliance matrix **C**, as 
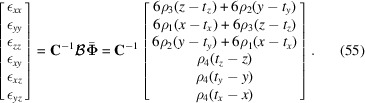
Numerical values of the constants ρ_1_, ρ_2_, ρ_3_, ρ_4_, *t*_*x*_, *t*_*y*_ and *t*_*z*_ are presented in Table 1 ▸.

The elasticity matrix for single-crystal tin was taken from Darbandi *et al.* (2013 ▸) (Table 2 ▸) and converted from Voigt notation to the used strain vector notation.

Parameters presented in Table 3 ▸ were used to define the experimental setup of the simulation.

The unit cell in Table 4 ▸ was used to define a strain-free lattice state.

**Figure 5:**
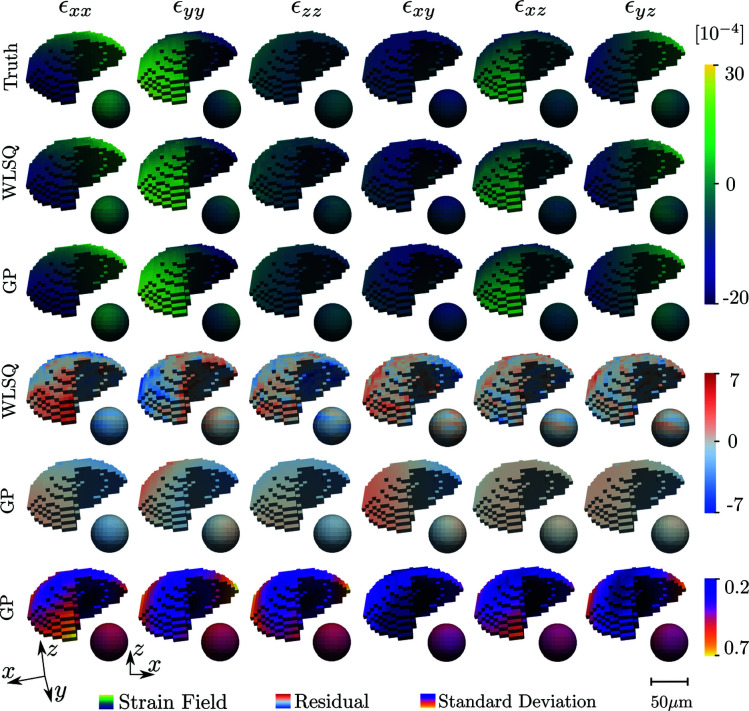
3D rendering of strain reconstructions for WLSQ and GP regression approaches. The top row defines the simulation ground truth as described in equation (55), with each column featuring a different strain component. The surface of the voxelated grain is presented, together with a pulled-out interior spherical cut centred at the grain centroid with a diameter of 50 µm. The corresponding coordinate systems are depicted in the bottom left of the figure. Three separate colormaps have been assigned to enhance contrast for the various fields. However, units of strain remain the same across plots (×10^−4^). The residual field is defined as the difference between the ground truth and the reconstructed strain field.

The generated diffraction patterns were analysed on a per-*z*-slice basis using *ImageD11* (Wright, 2005 ▸) to compute scattering vectors and average crystal orientations for each *z* slice. The grain shape was then reconstructed on the basis of the normalized diffraction peak intensities using filtered backprojection (Poulsen & Schmidt, 2003 ▸). Next, the diffraction data were converted to average directional strains, as described in Section 3, and input into the WLSQ and GP reconstruction methods. The final reconstructed strain tensor fields are illustrated together with simulation ground-truth and residual fields in Fig. 5 ▸. The corresponding root-mean-square errors (RMSEs), mean absolute errors (MAEs) and maximum absolute errors for the residual fields are given in Table 5 ▸.

**Figure 6:**
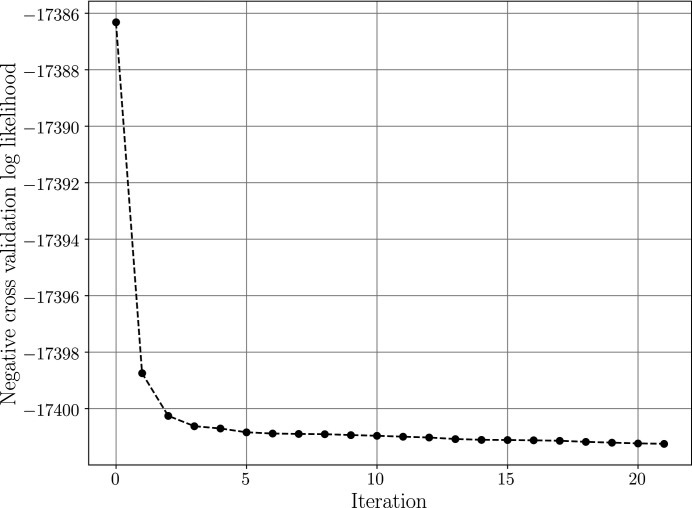
Negative cross-validation log likelihood reduction during hyperparameter optimization for the simulated grain presented in Figs. 4 ▸ and 5 ▸. Optimization was conducted using the L-BFGS-B algorithm as implemented in *SciPy* with a maximum of ten line-search steps per iteration. Gradients were computed using automatic differentiation as implemented in *PyTorch*.

Hyperparameters were optimized using the L-BFGS-B algorithm, as implemented in *SciPy* (Jones *et al.*, 2001 ▸), with a maximum of ten line-search steps per iteration. Gradients were computed using automatic differentiation as implemented in *PyTorch* (Paszke *et al.*, 2019 ▸). In the first optimization iteration all hyperparameters were uniformly set to the grain radius. The convergence of the optimization is displayed in Fig. 6 ▸. The smoothness constraints for the WLSQ in the *xy* plane were set to 2.5 × 10^−4^, limiting the maximum absolute difference in each strain tensor component between two neighbouring voxels [further details are provided by Henningsson *et al.* (2020 ▸)].

**Figure 7:**
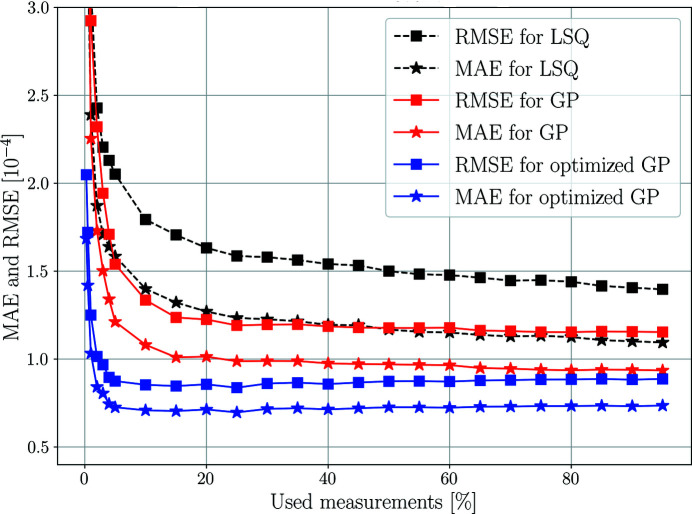
Average root-mean-square error (squares) and mean absolute error (stars) for the simulated grain presented in Figs. 4 ▸ and 5 ▸ as a function of used percentage of measurements. The performance of the Gaussian process regression (red and blue filled lines) is compared with that of the weighted least squares (black dashed lines). The RMSE and MAE were computed from the residual fields and averaged over the six reconstructed strain components to produce a scalar measure per reconstruction. Each point in the plot corresponds to a full 3D strain reconstruction using a random subset of the measured data.

To assess how well the two methods (WSLQ and GP) utilize data, the MAE and RMSE of the reconstructed strain fields, as a function of the number of input measurement integrals, has been investigated. By measurements we here refer to the integral values, *y*_*j*_, as defined in (24), together with their associated vectors (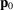, 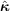, 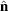). Measurements were permuted randomly and input into the WLSQ and GP reconstruction in initial sample sizes of 1, 2, 3, 4 and 5%, after which the sample size was increased in steps of 5% as indicated by the markers in Fig. 7 ▸. Since the GP hyperparameter optimization is a non­convex problem, the quality of any found local minima may vary between runs, and a better local minimum is not guaranteed with a larger measurement set owing to the different topology of the cost function. Thus, in order not to obscure the convergence rate of the GP method, we have selected to present results using fixed optimized hyperparameters found using 10% of the measurements as well as for non-optimized hyperparameters, set uniformly to the grain diameter. The resulting MAE and RMSE for the reconstructed residual fields were computed and averages over the six strain components were formed. The performance as a function of input measurements can be assessed by visual inspection of Fig. 7 ▸.

### 5.2. Embedded tin grain   

**Figure 8:**
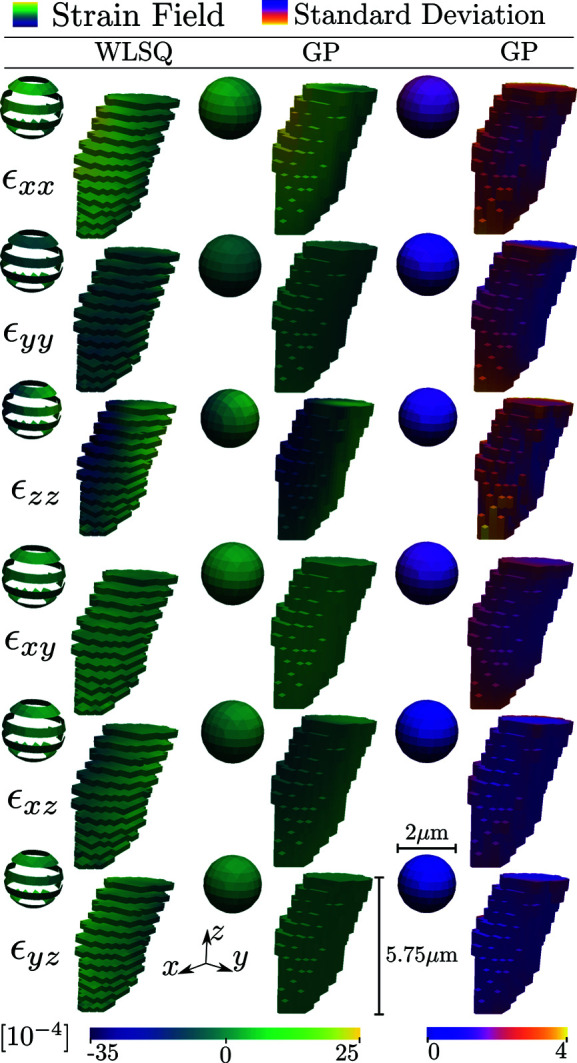
Reconstructed strain field using WLSQ (left column) and the GP method (middle column) of a columnar tin grain embedded within a polycrystalline sample. The rightmost column depicts the estimated uncertainty of the GP reconstruction. The 3D surface of the voxelated grain is presented together with a pull-out enlarged interior spherical cut with its centre at the grain centroid and a radius of 1 µm. Two separate colormaps have been assigned to enhance contrast for the various fields. However, the units of strain remain the same across plots (×10^−4^).

To further compare the GP and WLSQ reconstruction methods, analysis of a previously studied columnar tin grain has been included. This additional analysis further serves to show that the presented method is computationally feasible for state-of-the-art scanning 3DXRD data sets. Including hyperparameter optimization, the GP reconstruction was performed on a single CPU (Intel i7-8700K CPU @ 3.70 GHz) in 18 min and 9 s. As mentioned in Section 4.4, the computational complexity scales as 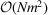, where *m* is the number of basis functions and *N* the number of measurements. The corresponding runtime using fixed precomputed hyperparameters was 3.5 s. The data for this example from Hektor *et al.* (2019 ▸) and the input experimental parameters are identical to those presented in Table 3 ▸ except for the beam size, which was 0.25 µm. Similarly, the relaxed lattice state was as defined in Table 4 ▸. In the original experiment, the X-ray beam was scanned across the *xy* plane, producing a space-filling map of measurements. However, owing to time constraints, the data were collected for every second *z* layer, as seen in the rightmost column of Fig. 8 ▸. The reader is referred to the original publication (Hektor *et al.*, 2019 ▸) for further information on the experimental setup, sample and preliminary data analysis.

As the GP method uses a nonlocal basis representation of the strain field, as defined in equation (39), interpolation between measured slices is an automatic feature of the method. For the WLSQ method, although some interpolation scheme could be selected, we have chosen to present the raw reconstructions. This also highlights the added benefit of the selected basis for the GP method. Hyperparameter optimization and smoothness constraints for the WLSQ method were applied and selected as in Section 5.1.

## 6. Discussion   

Comparison of the true and predicted fields in Fig. 5 ▸ for the two methods indicates that the reconstructions captured well the simulated input strain state. For all strain components in Table 5 ▸, both the RMSE and MAE are of the order of the expected experimentally limited strain resolution (10^−4^). We note, however, that the GP has consistently lower RMSE, MAE and maximum absolute errors in comparison with the WLSQ. The enhanced performance is attributed to the joint effect of the equilibrium prior, optimized correlation kernel and nonlocal basis selection.

The results of Table 5 ▸ indicate that, in general, the strain tensor *z* components enjoy more accurate reconstructions than the *xy* components. This observation is in line with previous work (Margulies *et al.*, 2002 ▸; Lionheart & Withers, 2015 ▸; Henningsson *et al.*, 2020 ▸) and is explained by the nonuniform sampling of strain taking place in scanning 3DXRD. The GP regression quantifies this phenomenon via the reconstructed standard deviation fields (Fig. 5 ▸, bottom row). Indeed the uncertainty in the predicted mean is elevated for the *xx* and *yy* components, which show similar patterns to the residual fields.

On the performance of the two methods, Fig. 7 ▸ indicates that fewer measurements are needed for the GP compared with the WLSQ approach whilst achieving a more accurate result. Little reduction in the RMSE and MAE is seen for the GP after about 50% of the measurements have been introduced (about 20% for the optimized GP version). This could imply that it is possible to retrieve approximations to the full strain tensor field from reduced scanning 3DXRD data sets. This could be attractive as scanning 3DXRD typically has time-consuming measurement sequences. From Fig. 7 ▸ it is also clear that the final errors in reconstruction will be nonzero. This is so because the error in reconstruction is made up of both bias and variance. While the variance can be reduced by adding more measurements, the bias is due to systematic errors arising from incorrect model assumptions such as the line integral approximation, the truncated covariance basis series expansion, the Taylor series expansion related to the strain measure, the directional approximation of 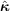 and possibly further unknown sources. Since the bias cannot be removed by adding more measurements, the reconstruction error will face a lower nonzero bound.

It is evident that the reconstructed fields have maximum uncertainties at the boundary of the grain, as can be seen from the cutout spheres of Figs. 5 ▸ and 8 ▸. The elevated standard deviation at the grain surface is explained by the tomographic measurement procedure, which has an increasing measurement density towards the grain centroid. Furthermore, as measurements do not exist outside of the grain, points lying on the grain surface will, in some sense, have a reduced number of points that they are correlated with. In addition to these effects, the selected line beam approximation may have an impact on the grain boundary errors. If the full 3D profile of the beam had been used instead, a higher number of scans that partially graze the grain boundary could have been included in the analysis, thus increasing the measurement density at the boundary. In the current model, if a scan has a geometric centre that does not intersect the grain, it has no impact on the reconstruction, even though the full 3D beam may have some overlap with the grain. The main challenge with using a full 3D beam profile, rather than the line approximation, is to maintain analytical expressions during integration of the partial derivatives of the basis functions over the illuminated domain.

The predicted strain field of the columnar tin grain of Fig. 8 ▸ shows similar patterns for the two regression methods. The uncertainty is again seen to be reduced on the interior of the grain, and the posterior standard deviation is of the order of the experimental strain resolution of 10^−4^. This validates the applicability of GP regression on real state-of-the-art scanning 3DXRD synchrotron data.

### 6.1. Outlook   

Two future potential improvements to strain predictions should be mentioned. Firstly, the selection of covariance function, although restricted to give a positive definite covariance matrix, is not unique; other selections may outperform the squared-exponential kernel used here. Secondly, for polycrystalline samples, additional prior knowledge of grain boundary strain could be extracted by considering the total sample grain map and that tractions must cancel on the interfaces [*i.e.* incorporating and extending the work of Hendriks, Gregg *et al.* (2019 ▸)]. Two challenges with this exist: (i) the uncertainty in reconstructed grain shapes leading to uncertainty in the interface normal and (ii) uncertainty in the per-point grain orientation leading to uncertainty in the grain compliance. The first of these challenges may be addressed by using near-field techniques (Viganò *et al.*, 2016 ▸) in conjunction with scanning 3DXRD to achieve higher-resolution grain maps.

## 7. Conclusions   

Intragranular strain estimation from scanning 3DXRD data using a Gaussian process is shown to provide a new and effective strain reconstruction method. By selecting a continuous differentiable Fourier basis for the Beltrami stress functions, a static equilibrium prior can be incorporated into the reconstruction, guaranteeing that the predicted strain field will satisfy the balance of both angular and linear momentum. The regression procedure results in a per-point estimated mean strain and per-point standard deviations, providing new means of estimating the per-point uncertainty of the reconstruction. Furthermore, the proposed method incorporates the spatial structure of the strain field by making use of a generic covariance function, optimized by maximizing the out-of-sample log likelihood. With the introduction of these three features, the equilibrium prior, the per-point uncertainty quantification and the optimized spatial smoothness constraints, the proposed regression method addresses weaknesses discussed in previously proposed reconstruction methods. Specifically, in comparison with a previously proposed weighted least-squares approach, it is found, from numerical simulations, that the Gaussian process regression consistently produces reconstructions with lower root-mean-square errors, mean absolute errors and maximum absolute errors across strain components. Moreover, it is shown that the reconstruction error as a function of the number of available measurements is reduced for the Gaussian process.

## Figures and Tables

**Figure 9:**
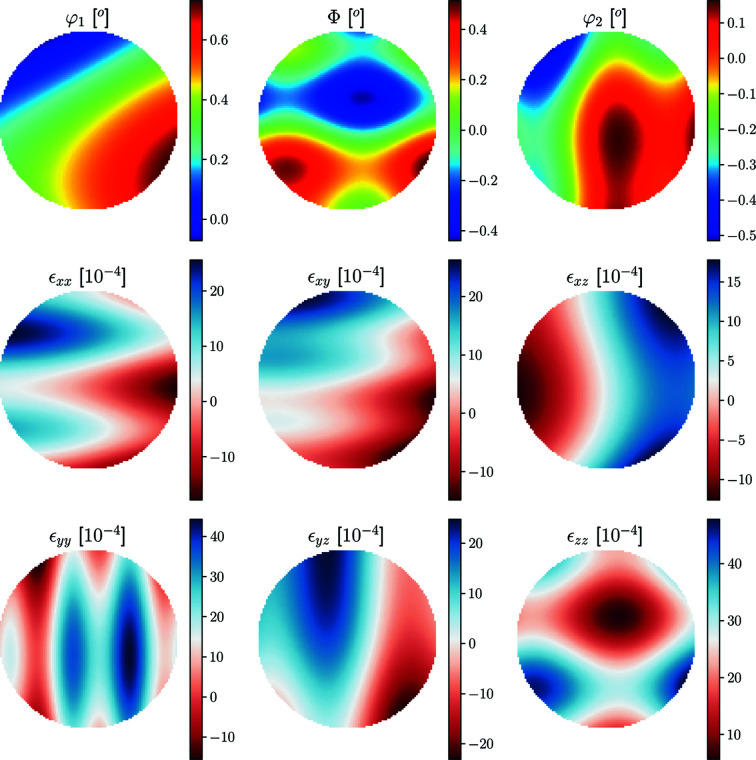
Typical Euler angle (top row) and strain (bottom and middle rows) fields generated by the stochastic model presented in equation (62). The presented fields exist on a spherical domain for which a central cut slice has been presented above (*z* = 0). The maximum difference parameters of the field were *A*_e_ = 1.4 and *A*_s_ = 75 × 10^−4^.

**Figure 10:**
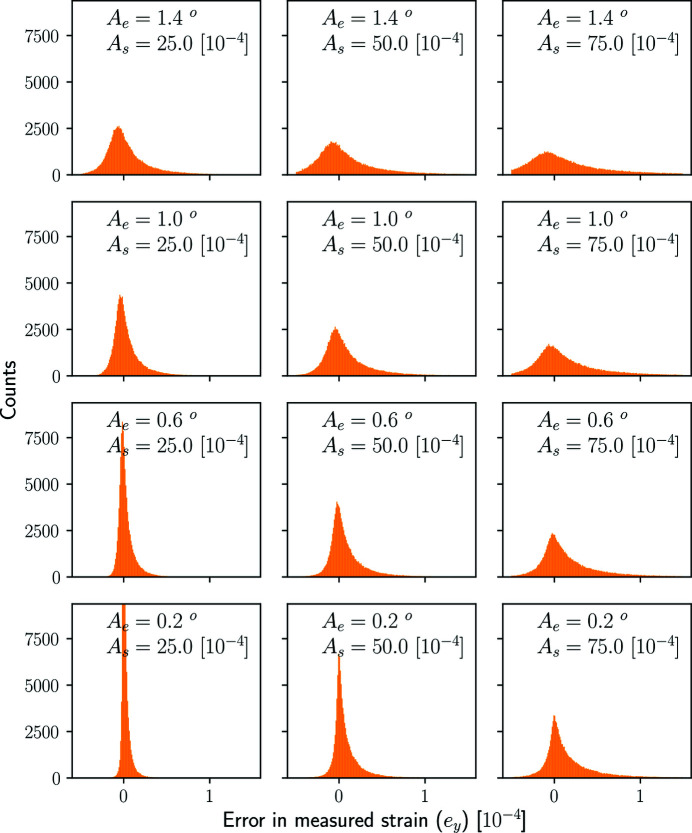
Absolute error (59) computed for the stochastic model defined through equations (60), (61) and (62). For each histogram 1000 random line integral measurements have been sampled from each of 100 spherical crystal states, resulting in a total of 100 000 data points per histogram. The maximum field difference in Euler angles and strain (*A*_e_ and *A*_s_) increases from bottom to top and left to right, respectively, as indicated by the figure labels. (Note that the maximum counts of the bottom-left plot have been clipped in order to facilitate equal axes between subplots while maintaining good visibility of the histograms.)

**Table 1:** Strain field parameters for diffraction simulation in units of µm

ρ_1_	ρ_2_	ρ_3_	ρ_4_	*t*_*x*_	*t*_*y*_	*t*_*z*_
100	100	100	1000	10	10	0

**Table 2:** Elasticity constants for single-crystal tin in units of GPa converted from Voigt notation as given by Darbandi *et al.* (2013 ▸)

*C*_11_	*C*_22_	*C*_33_	*C*_44_	*C*_55_	*C*_66_	*C*_12_	*C*_13_	*C*_23_
72.3	72.3	88.4	48.0	44.0	44.0	59.4	35.8	35.8

**Table 3:** Experimental parameters used in single-grain simulation, corresponding to the results presented in Fig. 5 ▸

Wavelength	0.22 Å
Sample-to-detector distance	163 mm
Detector pixel size	50 × 50 µm
Detector dimensions	2048 × 2048 pixels
Beam size	5 × 5 µm
ω rotation interval	[0, 180°]
Δω step length	1°
Maximum grain size in *x*	130 µm
Maximum grain size in *y*	130 µm
Maximum grain size in *z*	65 µm

**Table 4:** Relaxed reference lattice parameters

*a*	*b*	*c*	α	β	γ
5.81127 Å	5.81127 Å	3.17320 Å	90.0°	90.0°	90.0°

**Table 5:** Root-mean-square errors, mean absolute errors and maximum absolute errors for the residual fields presented in Fig. 5 ▸ The result of the Gaussian process regression is compared with the weighted least-squares fit (WLSQ). Values are unitless (strain) and on the same scale (10^−4^) as in Fig. 5 ▸.

	RMSE		MAE		Maximum absolute error	
Strain	GP	WLSQ	GP	WLSQ	GP	WLSQ
ε_*xx*_	1.322	2.076	1.101	1.737	2.791	7.42
ε_*yy*_	1.042	1.371	0.846	0.999	3.856	6.094
ε_*zz*_	0.887	1.489	0.769	1.157	1.914	6.736
ε_*xy*_	1.122	1.511	0.955	1.172	2.778	6.306
ε_*xz*_	0.24	1.04	0.198	0.798	1.506	4.85
ε_*yz*_	0.48	0.958	0.399	0.742	1.34	4.652

## Appendix A Error related to measurement approximations

To demonstrate the accuray of the approximations made in (15), we investigate the error associated with the fact that both strain and crystal orientation may vary along the ray path. To do this we must consider that the strain computed from (15) is further assigned to planes with approximate normals given by (17). Thus, there is a twofold error source to capture in the following analysis, arising partly from the integrated Taylor series expansion,

and partly from assigning the average strain value, *y*, to an incorrect plane normal,

which in reality is not fixed but warps across the crystal [].

To compute the error in strain we consider first the true average strain for a single line-integral measurement, *y*_true_, existing in a fixed direction, :

where the average strain tensor 〈**ε**_ω_〉 is unknown from the experiment. We stress that we are interested in the true strain for a fixed  since this is the normal that the approximation of (56) will eventually be assigned to. The sought absolute error now becomes

For a given set of Miller planes, strain field, Euler angle field, reference unit cell and integration domain , equation (59) can be evaluated. To do so, two integrations must be performed, yielding individually 〈**G**_ω_〉 and 〈**ε**_ω_〉. In the following we attempt to characterize (59) for a fixed reference unit cell (Table 4 ▸) while letting the remaining parameters vary according to a stochastic model described below. The goal is to study the distribution of the absolute errors as a function of increasing levels of intragranular strain and mosaicity to understand the limitations of the proposed measurement approximations.

We consider a spherical grain of fixed radius *R*_0_ = 1.0 centred at the origin and define a random integration domain, , as

where *L* is determined by the sphere line intersection, **p**_0_ is a random uniform point on the sphere surface and  is a unit vector also drawn from a random uniform distribution denoted . We further define **G**_*hkl*_ as

where the distribution has been limited to the interval [−7, 7] to represent typical scanning 3DXRD data sets. Pseudo-random strain and orientation fields are introduced by constructing random samples of superimposed Fourier waves:

where the two scale parameters *A*_s_ and *A*_e_ regulate the maximum difference between any two points within the field and allow for the strain field (scaled by *A*_s_) and orientation field (scaled by *A*_e_) to vary on different scales simultaneously. The necessary normalizing factors  were computed by sampling the fields on equidistant grids of ∼1000 points and selecting the maximum absolute value. Typical fields generated by the model can be seen in Fig. 9 ▸.

Using the above stochastic model we have performed repetitive sampling of 1000 line integral measurements for each of 100 grain states while letting *A*_s_ and *A*_e_ successively increase between samples. The results of this analysis are presented in the histograms of Fig. 10 ▸, where each histogram corresponds to a total of 100 000 data points (100 × 1000) and a fixed maximum field variation (*A*_s_, *A*_e_). The average strain 〈**ε**_ω_〉 and diffraction vector 〈**G**_ω_〉 involved in (59) were computed by first-order numerical integration using a total of 20 integration points along each domain . The reference orientation matrix, **U**_0_, was computed by averaging over ∼1000 equally spaced points of the sphere.

The results of Fig. 10 ▸ show that the error in (59) increases with the heterogeneity of both orientation and strain state. For small strains (≤50 × 10^−4^) and moderate mosaic spreads (≤1°) the largest errors are a few times that of the experimental resolution limit (10^−4^) and the bulk (>95%) of measurements are contained within [±10^−4^] units of strain. For samples featuring larger mosaicity (>1.0°) and strain variation (>75 × 10^−4^) the approximation starts to break down. At such elevated levels of deformation, however, one has to first consider if the small-strain approximation made in (7) is still valid.

Specifically, for the synthetic data set presented in this paper (Figs. 4 ▸ and 5 ▸) we conclude that the input strain and orientation fields will give rise to a negligible error, *e*_*y*_ < 10^−4^. Likewise for the tin grain presented in Fig. 8 ▸, on the basis of the observed diffraction peak spread in ω, the mosaic spread is <1.0° and thus *e*_*y*_ is negligible.
